# Asymbiotic Seed Germination and In Vitro Propagation of the Thai Rare Orchid Species; *Eulophia bicallosa* (D.Don) P.F.Hunt & Summerh.

**DOI:** 10.3390/plants14142212

**Published:** 2025-07-17

**Authors:** Thanakorn Wongsa, Jittra Piapukiew, Kanlaya Kuenkaew, Chatchaya Somsanook, Onrut Sapatee, Julaluk Linjikao, Boworn Kunakhonnuruk, Anupan Kongbangkerd

**Affiliations:** 1 Plant Tissue Culture Laboratory, Program in Biology, Faculty of Science and Technology, Kamphaeng Phet Rajabhat University, Kamphaeng Phet 62000, Thailand; thanakorn_wo@kpru.ac.th; 2 Department of Botany, Faculty of Science, Chulalongkorn University, Pathum Wan, Bangkok 10330, Thailand; jittra.k@chula.ac.th; 3 Plant Tissue Culture Research Unit, Department of Biology, Faculty of Science, Naresuan University, Muang, Phitsanulok 65000, Thailand; kanlayahha@hotmail.com (K.K.); chuchchayas59@email.nu.ac.th (C.S.); onrut_nu51411546@hotmail.com (O.S.); julalukl62@nu.ac.th (J.L.); bowornk@nu.ac.th (B.K.); 4 Center of Excellence in Research for Agricultural Biotechnology, Naresuan University, Muang, Phitsanulok 65000, Thailand

**Keywords:** terrestrial orchid, media composition, organic supplements, plant growth regulators

## Abstract

An efficient in vitro propagation protocol for *Eulophia bicallosa* was developed using asymbiotic seed germination and protocorm proliferation. The effect of light on seed germination and development was evaluated on Vacin and Went (VW) medium under five conditions: darkness, white, green, red, and blue light for 24 weeks. Blue and red light significantly accelerated seed development, allowing progression to stage 5 within 24 weeks. For protocorm proliferation, six semi-solid culture media were tested. Half-strength Murashige and Skoog (½MS) medium yielded the best results after 8 weeks, producing the highest numbers of shoots (1.0), leaves (1.1), and roots (4.2) per protocorm, with 100% survival. The effects of organic additives were also evaluated using coconut water and potato extract. A combination of 200 mL L^−1^ coconut water and 50 g L^−1^ potato extract enhanced shoot formation (1.7 shoots), while 150 mL L^−1^ coconut water with 50 g L^−1^ potato extract increased both leaf (1.9) and root (8.8) numbers. The effects of cytokinins (benzyladenine (BA), kinetin (6-furfurylaminopurine), and thidiazuron (TDZ)) and auxins (indole-3-acetic acid (IAA), α-naphthalene acetic acid (NAA), indole-3-butyric acid (IBA), and 2,4-dichlorophenoxyacetic acid (2,4-D)) were investigated using ½MS medium supplemented with each plant growth regulator individually at concentrations of 0, 0.1, 0.5, 1.0, and 2.0 mg L^−1^. Among the cytokinins, 0.1 mg L^−1^ BA produced the highest survival rate (96%), while 1.0 mg L^−1^ BA induced the greatest shoot formation (93%, 2.3 shoots). Among the auxins, 0.1 mg L^−1^ IAA resulted in the highest survival (96%), and 1.0 mg L^−1^ IAA significantly enhanced root induction (4.2 roots per protocorm). Acclimatization in pots containing a 1:1:1 (*v*/*v*) mixture of pumice, sand, and soil resulted in 100% survival. This protocol provides a reliable and effective approach for the mass propagation and ex situ conservation of *E*. *bicallosa*.

## 1. Introduction

*Eulophia* R.Br. is a diverse genus comprising approximately 281 orchid species found across tropical and subtropical regions [1]. Most species are terrestrial, with rhizomes growing above or below ground, and produce shoots and inflorescences with varied floral morphology, fragrance, and phenology [2,3]. Several species are valued ornamentally and occasionally cultivated. Many also hold ethnobotanical significance, particularly in traditional medicine and as food sources [4,5]. *Eulophia bicallosa* (D. Don) P.F. Hunt & Summerh. is a terrestrial orchid with subterranean rhizomes and pale green to cream-colored flowers featuring a purplish labellum [6]. It is naturally distributed across India (Assam and Sikkim), Bhutan, Nepal, New Guinea, the Philippines, Queensland (Australia), and Thailand [7,8,9,10]. In Thailand, it has been recorded in only two freshwater swamp habitats in Nakhon Sawan and Rayong provinces [11]. Natural populations of *E*. *bicallosa* are in decline due to habitat destruction, illegal collection, and overharvesting [12]. Its propagation is further constrained by mycorrhizal dependence for seed germination and slow rhizome multiplication [13]. As a result, the species is listed under CITES Appendix II [14], underscoring the need for effective propagation protocols to support conservation. In vitro culture offers a practical alternative, complementing in situ efforts that are often limited by environmental constraints. Asymbiotic seed germination is widely used for large-scale propagation and germplasm conservation in orchids [15,16,17]. Although in vitro propagation has been reported in related species such as *E*. *cucullate*, *E*. *streptopetala*, and *E*. *petersii* [18], no studies have addressed *E*. *bicallosa*, highlighting the need for a species-specific protocol. Successful in vitro germination depends on multiple factors, including culture medium composition, organic additives, plant growth regulators, and light conditions [17,19,20]. Light quality, particularly spectral composition, strongly influences seed germination and protocorm development in orchids [21,22,23]. For instance, white and deep-red Light Emitting Diode (LED) promote protocorm development in *Epidendrum fulgens* [24], while blue and red LEDs improve PLB induction in *Phalaenopsis pulcherrima* [25]. In *Vanilla planifolia*, red LED light results in the highest germination rates [26], demonstrating species-specific responses. Culture media also play a critical role by providing essential nutrients [27,28]. Commonly used media include Murashige and Skoog (MS) [29], Vacin and Went (VW) [30], Basic Medium-1 (BM-1) [31], Knudson C (KC) [32], and Malmgren Modified Medium (MM) [33]. Requirements vary by species: MS supports germination in *Eulophia flava* [34]; New Dogashima (ND) medium is optimal for *Vanilla siamensis* [35] and *Gastrochilus japonicus* [36], and MM supports *Orchis simia* and *Ophrys schulzei* [37]. Organic additives enhance in vitro development. Substances such as coconut water, banana and potato homogenates, tomato and pineapple juice, yeast extract, and papaya extract have shown positive effects [20,38]. For instance, pineapple juice and coconut water promote seedling growth in *Laelia rubescens* [39], while banana powder and potato extract enhance germination in *Doritis pulcherrima* [40]. Plant growth regulators, particularly cytokinins such as benzyladenine (BA), kinetin (6-furfurylaminopurine), and thidiazuron (TDZ) and auxins including indole-3-acetic acid (IAA), α-naphthalene acetic acid (NAA), indole-3-butyric acid (IBA) play crucial roles in shoot and root induction, cell division, and organogenesis [41,42,43]. These have been effectively applied in various orchids, including *Orchis militaris*, *Anacamptis pyramidalis*, *Geodorum densiflorum*, and *Paphiopedilum villosum* [44,45,46,47]. In *Eulophia*, BA promotes shoot formation in *E*. *dabia* and *E*. *graminea* [48], while IAA and NAA enhance root and rhizome development in *E*. *streptopetala* and *E*. *flava* [34,49]. Acclimatization, the final stage of micropropagation, is critical for transitioning plantlets to ex vitro conditions. For rhizomatous orchids like *Eulophia*, substrate selection such as clay, sand, soil, peat, or vermiculite is crucial for survival and further development [17,48]. To date, no studies have focused on the propagation or conservation of *E*. *bicallosa*. This study presents the first comprehensive investigation of its in vitro seed germination, seedling development, and acclimatization. The findings contribute to ex situ conservation, support population restoration, and provide a foundation for propagating other threatened *Eulophia* species.

## 2. Materials and Methods

### 2.1. Plant Materials and Surface Sterilization of Capsules

**Figure 1:**
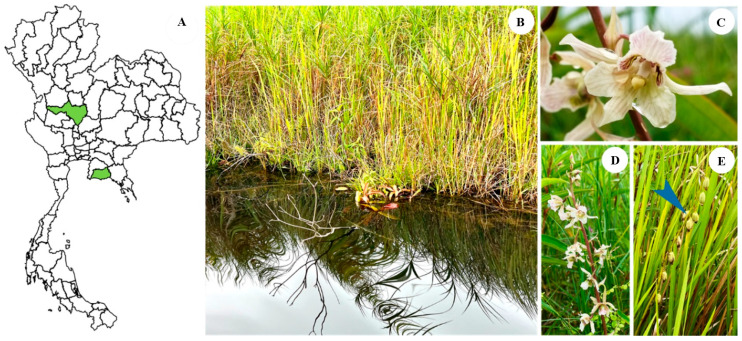
Distribution areas of *E*. *bicallosa* in Thailand, including the Lower North (Nakhon Sawan province) and Southeast (Rayong province) (**A**). Freshwater swamp habitat in Rayong province (**B**), flowering stage (**C**), fully bloomed inflorescence (**D**), and fruit set from naturally pollinated flowers (**E**). All photographs were taken in August.

Mature capsules of *E*. *bicallosa*, collected 12–16 weeks after natural pollination in mid-August from wetland habitats in Rayong province (Figure 1), were used in this study. The experiment was conducted at the Plant Tissue Culture Research Unit, Department of Biology, Faculty of Science, Naresuan University, Thailand. The capsules were first washed under running tap water for 15 min, followed by surface sterilization with 15% (*v/v*) sodium hypochlorite (NaClO) solution (Kao Industrial Co., Ltd., Chonburi, Thailand) for 15 min. After sterilization, they were rinsed three times with sterile distilled water and air-dried in a laminar flow cabinet at 25 ± 2 °C for 5 min. Once dried, the capsules were carefully dissected using a sterile surgical blade to expose the seeds. The seeds were then collected, combined, and thoroughly mixed for subsequent experiments.

### 2.2. Effect of Light Quality on the Germination and Development of E. bicallosa Seeds

All seeds obtained from the sterilization process were transferred to test tubes containing 20 mL of semi-solid Vacin and Went (VW) medium [30], supplemented with 20 g L^−1^ sucrose and 7.5 g L^−1^ agar, with the pH adjusted to 5.2. The cultures were maintained under controlled conditions at 25 ± 2 °C and exposed to various light treatments for 12 h per day. These treatments included warm-white light-emitting diode (LED) strip illumination (peak wavelength 565–573 nm; 40 μmol m^−2^ s^−1^), green light (526 nm; 10 μmol m^−2^ s^−1^), red light (660 nm; 10 μmol m^−2^ s^−1^), blue light (440–450 nm; 10 μmol m^−2^ s^−1^) (Leewattana Products Co., Ltd., Bangkok, Thailand), and complete darkness. For each treatment, seeds were cultured in four test tubes. For each treatment, seeds were cultured in four test tubes. In each replicate, 100 seeds were randomly selected and counted, with four replicates per treatment. The germination process was monitored using a stereo microscope, and the seed germination rate and developmental stages were recorded weekly over a period of 24 weeks. The developmental stages of *E*. *bicallosa* germination were classified into five stages, adapted from the method described by [50] (Table 1).

### 2.3. Effect of Different Culture Media on the Growth and Development of Protocorms

**Figure 2:**
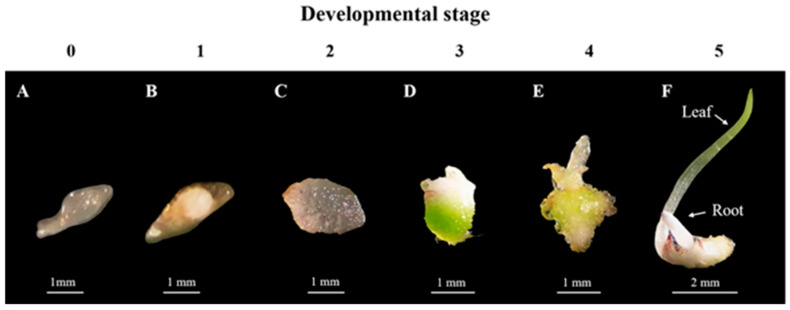
In vitro asymbiotic seed germination and seedling development of *E*. *bicallosa*: Stage 0, hyaline embryo with an intact testa after 3 weeks (**A**); Stage 1, swollen embryo with a ruptured testa and rhizoids present after 4 weeks (**B**); Stage 2, appearance of the protomeristem at 8 weeks (**C**); Stage 3, formation of a rhizome-like structure at the base of the protomeristem in the protocorm at 12 weeks (**D**); Stage 4, continued enlargement of the rhizome-like structure and/or emergence of the first leaf from the rhizome-like structure at 20 weeks (**E**); Stage 5, fully developed leaf from shoot-derived protocorms after 24 weeks of culture (**F**).

To evaluate the influence of different culture media, six types of media were tested: half-strength (½MS) and full-strength (MS) Murashige and Skoog medium [29], Vacin and Went (VW) medium [30], Basic Medium-1 (BM-1) for terrestrial orchids [31], Knudson C (KC) medium [32], and Malmgren modified terrestrial orchid (MM) medium [33] (Phytotechnology Laboratories, Lenexa, KS). The pH values of the media were adjusted as follows: ½MS and MS to 5.8, VW and KC to 5.2, BM-1 to 5.5, and MM to 5.75. All media were solidified with 7.5 g L^−1^ agar (Pearl Mermaid Agar, Patanasin Enterprise Ltd., Part, Bangkok, Thailand). Semi-solid media (10 mL) were dispensed into clear glass bottles with a volume of approximately 59.15 mL (2 oz) and autoclaved at 121 °C for 15 min. Protocorms of *E*. *bicallosa* at stage 3, with an approximate diameter of 1–2 mm in size (Figure 2D) and derived from seed germination on semi-solid VW medium under dark conditions, were used as explants. Two protocorms were placed in each bottle and cultured under controlled conditions with a 12-h light cycle (light intensity of 20 μmol m^−2^ s^−1^) provided by cool-white fluorescent tubes at 25 ± 2 °C for 8 weeks. The experiment was conducted in triplicate, with each replicate consisting of 30 protocorms. After 8 weeks of culture, data on survival rate, percentage of shoot, root, and rhizome formation, as well as the number of shoots, leaves, and roots, were recorded.

### 2.4. Effect of Coconut Water and Potato Extract on the Growth and Development of Protocorms

To determine the effect of organic additives, protocorms of *E*. *bicallosa* 1–2 mm in size were culture on ½MS medium supplemented with coconut water (CW) and potato extract (PE) in various concentrations ratio (CW: 0, 100, 150, 200 mL L^−1^ and PE: 0, 5, 10, 15 mL L^−1^). The culture medium was solidified with 7.5 g L^−1^ agar, with the pH adjusted to 5.8 and then semi-solid media were dispensed a 10 mL into 2 oz clear glass bottles and autoclaved at 121 °C for 15 min. Each bottle contained two protocorms then they were transferred to culture under controlled conditions with a 12-h light cycle (intensity 20 μmol m^−2^ s^−1^), provided by cool-white fluorescent tubes, at 25 ± 2 °C for 12 weeks. The experiment was conducted in triplicate, with each repetition consisting of 30 protocorms. After 12 weeks, survival rate, shoot formation, root formation, rhizome formation, and the number of shoots, leaves, roots, and rhizomes were observed.

### 2.5. Effect of Cytokinins on the Growth and Development of Protocorms

Two individual protocorms, each 1–2 mm in size, were cultured on ½MS medium supplemented with varying concentrations of benzyladenine (BA), kinetin (6-furfurylaminopurine), and thidiazuron (TDZ), each applied individually at 0, 0.1, 0.5, 1.0, and 2.0 mg L^−1^ (Phyto Technology Laboratories). The medium was solidified with 7.5 g L^−1^ agar and adjusted to pH 5.8. Subsequently, 10 mL of the semi-solid medium was dispensed into 2 oz clear glass bottles and sterilized by autoclaving at 121 °C for 15 min. Protocorms were cultured under controlled conditions with a 12 h photoperiod (light intensity: 20 μmol m^−2^ s^−1^), provided by cool-white fluorescent tubes, at 25 ± 2 °C for 12 weeks. The experiment was conducted in triplicate, with each replicate consisting of 30 protocorms. After 12 weeks of culture, survival rate, callus induction, shoot formation, root formation, rhizome formation, and the numbers of shoots, leaves, roots, and rhizomes were recorded.

### 2.6. Effect of Auxins on the Growth and Development of Protocorms

Two individual protocorms, each 1–2 mm in size, were cultured on ½MS medium supplemented with varying concentrations of indole-3-acetic acid (IAA), α-naphthalene acetic acid (NAA), indole-3-butyric acid (IBA), and 2,4-dichlorophenoxyacetic acid (2,4-D), each applied individually at 0, 0.1, 0.5, 1.0, and 2.0 mg L^−1^ (Phyto Technology Laboratories). The medium was solidified with 7.5 g L^−1^ agar, adjusted to pH 5.8, and 10 mL of the semi-solid medium was dispended into 2 oz clear glass bottles. The media were sterilized by autoclaving at 121 °C for 15 min. Cultures were maintained under controlled conditions with a 12-h light cycle (light intensity: 20 μmol m^−2^ s^−1^), provided by cool-white fluorescent tubes, at 25 ± 2 °C for 12 weeks. The experiment was conducted in triplicate, with each replicate consisting of 30 protocorms. After 12 weeks of culture, the survival rate, percentages of callus induction, shoot formation, root formation, and rhizome formation, as well as the numbers of shoots, leaves, roots, and rhizomes, were recorded.

### 2.7. Greenhouse Acclimatization

Healthy plantlets, 7–10 cm in height with 1–2 leaves and 2–5 roots (each root 2–3 cm in length), were randomly selected from 12-week-old cultures grown on ½MS medium supplemented with either 1.0 mg L^−1^ BA or 0.1 mg L^−1^ IAA. A total of 50 plantlets per treatment were transplanted to assess survival during acclimatization. Roots were thoroughly rinsed with tap water to remove any residual agar. One plantlet was then transferred to an individual plastic pot (5-inch diameter) containing a mixture of pumice, sand, and loamy soil in a 1:1:1 (*v*/*v*) ratio. Transplantation was followed by maintenance under greenhouse conditions, where the average temperature was 27–28 °C and relative humidity was maintained at 80–85%. A misting system was used to irrigate the plants twice per day. During the 8-week acclimatization period, no additional plastic or transparent covering was used. The survival rate was recorded at the end of the acclimatization period.

### 2.8. Experimental Design and Data Analysis

The experiment was performed in a completely randomized design (CRD) with three replications. The differences of mean in among the treatments were analyzed using the one-way analysis of variance (ANOVA) test and compared using Duncan’s Multiple Range Tests (DMRT) at *p* ≤ 0.05.

## 3. Results

### 3.1. Effect of Light Quality on Seed Germination and Development

The effect of light on seed germination and development of *E*. *bicallosa* was evaluated on VW medium under five light conditions: darkness, white, green, red, and blue, over a 24-week period. No development was observed during the first three weeks (stage 0) (Figure 2A). By week 4, embryos swelled and emerged from the seed coat (stage 1) (Figure 2B), followed by protomeristem formation (stage 2) (Figure 2C). By week 12, seeds in all light treatments reached stage 3, characterized by the formation of rhizome-like bases and scale leaves (Figure 2D). At week 20, green, red, and blue lights further advanced development to stage 4, indicated by rhizome enlargement and true leaf emergence (Figure 2E). By week 24, only seeds under red and blue light reached stage 5, marked by true leaf formation and root development (Figure 2F).

**Figure 3:**
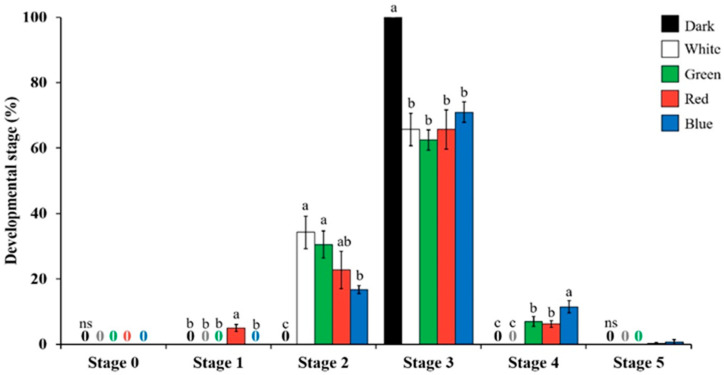
Developmental stages of asymbiotic germination and seedling growth of *E*. *bicallosa* under dark, white, green, red, and blue light after 24 weeks. Results represent the mean of four replicates (100 seeds per replicate). Error bars indicate the standard error (SE), and different letters on each bar denote significant differences at *p* ≤ 0.05 according to DMRT. ns, no significance.

In terms of developmental stage percentages by week 24, red light accelerated initial germination (stage 1) with a significantly higher rate (5.0%) compared to other treatments (Figure 3). Stage 2 development was most pronounced under white (34.3%) and green (30.5%) light, followed by red (22.8%) and blue (16.8%), and was absent in darkness. White and green light significantly promoted progression to stage 2 compared to darkness and blue light. Stage 3 development was similar across all light treatments (62.5–71.0%) but significantly lower than under dark conditions, where all seeds remained at stage 3 (100.0%), indicating developmental arrest in the absence of light. In contrast, exposure to colored light promoted progression to more advanced stages, while no stage 4 development was observed under dark and white conditions. Blue light induced the highest percentage of stage 4 development (11.5%), which was significantly greater than that under green (7.0%) and red (6.3%) light. The final developmental stage (stage 5) was observed only under red (0.3%) and blue (0.8%) light treatments, however, these differences were not statistically significant. Seeds exposed to other light conditions mostly remained at stages 3.

### 3.2. Effect of Culture Media on Survival and Morphological Responses of E. bicallosa Protocorms

The effects of different culture media on the survival, shoot, and root formation, rhizome development, and callus induction of *E*. *bicallosa* protocorms were assessed after 12 weeks of in vitro culture (Table 2). Protocorm survival was highest on ½MS medium (100.0%), although there was no statistically significant difference among treatments. In contrast, the lowest survival rates were recorded on VW and MM media (86.7%). Shoot formation was most pronounced on ½MS (93.3%), which was significantly higher than on all other media. Meanwhile, MM medium did not support any shoot formation.

Similarly, root formation was also highest on ½MS (96.7%), with statistically significant differences observed among treatments, while VW exhibited the lowest rooting percentage (40.0%). Rhizome development was most effective on ½MS (100.0%), whereas BM-1 induced only minimal rhizome formation (3.3%). Furthermore, callus formation occurred exclusively on MS medium (26.7%), which was significantly higher than on all other media, where no callus was observed.

### 3.3. Effect of Culture Media on the Number of Shoots, Leaves, Roots, and Rhizomes in E. bicallosa Protocorms

**Figure 4:**
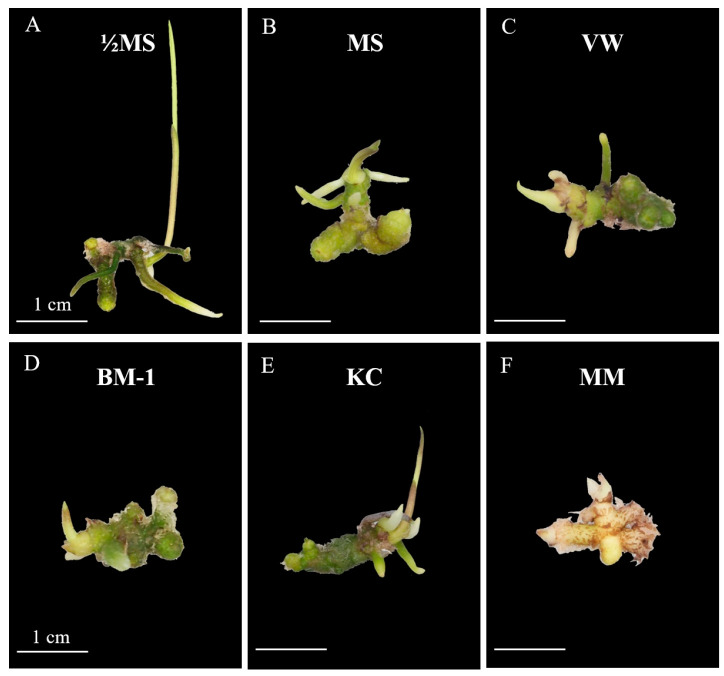
Growth and development of *E*. *bicallosa* protocorms after 8 weeks of culture on different media: (**A**) half-strength MS (½MS), (**B**) full-strength MS (MS), (**C**) VW, (**D**) BM-1, (**E**) KC, and (**F**) MM.

The effect of different culture media on the number of shoots, leaves, roots, and rhizomes in *E*. *bicallosa* protocorm was evaluated after 8 weeks of in vitro culture (Table 3 and Figure 4). Protocorms cultured on ½MS medium produced the highest number of shoots (1.0 shoot per protocorm) and leaves (1.1 leaves per protocorm), with statistically significant differences compared to all other treatments. In contrast, no shoot or leaf formation was observed on BM-1 and MM media. Root formation was also greatest on ½MS medium (4.2 roots per protocorm), significantly surpassing all other treatments. In contrast, BM-1 resulted in the lowest number of roots (0.6 root per protocorm). Rhizome formation ranged from 1.4 to 2.3 rhizome per protocorm across all treatments, with no statistically significant differences among the media. The highest mean rhizome number was recorded on BM-1 (2.3 rhizomes per protocorm), followed closely by VW (2.2 rhizomes per protocorm).

### 3.4. Effect of Coconut Water and Potato Extract on Survival, Shoot Formation, Root Formation, and Rhizome Development in Protocorms

The effects of coconut water (CW) and potato extract (PE) on the survival and development of protocorms are presented in Table 4. All treatments resulted in high survival rates, ranging from 86.7% to 100%. Regarding shoot formation, the highest rate (80.0%) was observed in ½MS medium supplemented with 25 g L^−1^ PE alone, followed by treatments with 50 g L^−1^ PE and a combination of 150 mL L^−1^ CW + 50 g L^−1^ PE (76.7%). However, increasing CW concentrations, particularly up to 200 mL L^−1^, led to a significant decline in shoot formation, with the rate dropping to 30.0%. Root formation was most pronounced in media containing 25 or 50 g L^−1^ PE without CW, achieving the highest rate of 90.0%. In contrast, media supplemented with CW alone at 100 or 200 mL L^−1^ showed significantly reduced root formation (36.7%), less than half of that observed with PE treatments (90.0%). Rhizome formation ranged from 76.7% to 100%. The highest rhizome formation rate (100%) was recorded in three treatments: 0 mL L^−1^ CW + 50 g L^−1^ PE, 150 mL L^−1^ CW + 25 g L^−1^ PE, and 50 mL L^−1^ CW + 100 g L^−1^ PE. However, increasing CW concentrations combined with high PE levels led to a decrease in rhizome formation, with the lowest rate (76.7%) observed in the treatment containing 200 mL L^−1^ CW + 100 g L^−1^ PE.

### 3.5. Effect of Coconut Water and Potato Extract on Shoots, Leaves, Roots, and Rhizomes Induction in Protocorms

**Figure 5:**
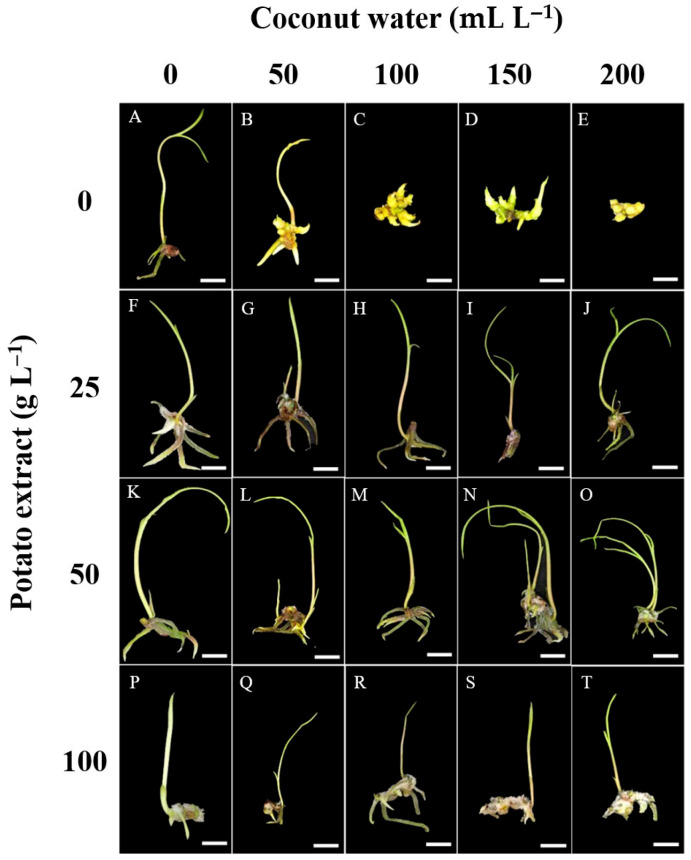
Protocorm development of *E*. *bicallosa* on ½MS medium supplemented with different combinations of coconut water (CW) and potato extract (PE) after 12 weeks of culture. (**A**) Slim shoots on medium without CW and PE; (**B**) plump shoots with PE alone; (**C**–**E**) multiple dwarf shoots with CW alone; (**F**–**J**) complete plantlets with multiple shoots, leaves, roots, and rhizomes on medium supplemented with 25 g L^−1^ PE and varying CW ratios; (**K**–**O**) similar development on medium with 50 g L^−1^ PE; (**P**–**T**) enhanced rhizome formation on medium with higher PE concentration. Scale bar = 1 cm.

The number of shoots, leaves, roots, and rhizomes in *E*. *bicallosa* protocorms are presented in Table 5 and Figure 5. Shoot formation increased with CW concentration, peaking at 1.7 shoots per explant in the 200 mL L^−1^ CW + 50 g L^−1^ PE treatment, while the lowest (0.6 shoots) was observed with 100 g L^−1^ PE alone. Leaf formation followed a similar pattern. The highest number of leaves (1.9 leaves per explant) occurred with 150 mL L^−1^ CW + 50 g L^−1^ PE. Moderate CW (50–200 mL L^−1^) combined with 25 g L^−1^ PE promoted leaf development (1.4–1.8 leaves per explant), whereas high PE (100 g L^−1^), particularly without CW, suppressed it (0.6 leaves per explant). Root development was most enhanced by 150 mL L^−1^ CW + 50 g L^−1^ PE (8.8 roots per explant). Treatments with 50–200 mL L^−1^ CW and 25–50 g L^−1^ PE yielded 6.6–7.3 roots per explant, while 100 g L^−1^ PE alone significantly reduced root formation (2.3 roots per explant). Rhizome formation ranged from 1.2 to 2.8 rhizomes per explant, with the highest observed in 150 mL L^−1^ CW without PE and the lowest in the control lacking both CW and PE.

### 3.6. Effect of Cytokinins on Survival Rate, Callus Induction, Shoot and Root Formation, and Rhizome Development in Protocorms

After 12 weeks of culture, the highest survival rate (96.0%) was observed in ½MS medium supplemented with 0.1 mg L^−1^ BA, significantly higher than the control (73.0%) and treatments with 2.0 mg L^−1^ BA, 2.0 mg L^−1^ kinetin, and 0.5–2.0 mg L^−1^ TDZ. Kinetin treatments also improved survival (83.3–89.7%), though less effectively than BA (Table 6). Callus formation was absent in the control, BA, and kinetin treatments but was significantly induced by TDZ. The highest callus induction (93.3%) occurred at 1.0 mg L^−1^ TDZ, with similar rates (90.0%) at 0.1, 0.5, and 2.0 mg L^−1^ TDZ. BA was the most effective cytokinin for shoot formation, with a peak at 1.0 mg L^−1^ (93.0%), followed by 0.5 mg L^−1^ (92.0%), both significantly higher than the control and TDZ treatments. Kinetin also promoted shoot formation (83.3–88.0%) across all concentrations, while TDZ completely inhibited shoot formation (Table 6). Root formation was highest with 0.1 mg L^−1^ kinetin (67.0%), followed by 1.0 mg L^−1^ BA (62.7%). However, root formation declined at higher BA concentrations (35.3% at 2.0 mg L^−1^). TDZ did not induce root formation at any concentration. Rhizome formation was highest with 0.1 mg L^−1^ BA (81.3%), comparable to 0.5 mg L^−1^ BA and the control. In contrast, TDZ failed to induce rhizome formation (Table 6).

### 3.7. Effect of Cytokinins on the Number of Shoots, Leaves, Roots, and Rhizomes

**Figure 6:**
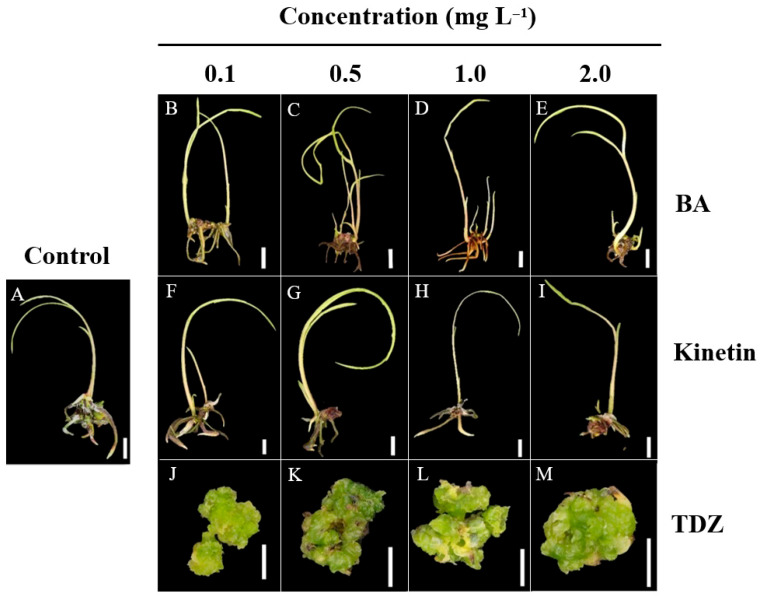
Effects of cytokinins on *E*. *bicallosa* protocorm development after 12 weeks of culture on ½MS medium supplemented with BA, kinetin, or TDZ at concentrations of 0.1, 0.5, 1.0, and 2.0 mg L^−1^. (**A**) Control (no cytokinin); (**B**–**E**) BA enhanced shoot formation; (**F**–**I**) kinetin promoted shoot elongation and root development; and (**J**–**M**) TDZ induced callus formation. Scale bar = 1 cm.

The number of shoots, leaves, roots, and rhizomes in *E*. *bicallosa* protocorms was significantly affected by cytokinin type and concentration (Table 7 and Figure 6). The highest shoot number (2.3 shoots per protocorm) was observed with 1.0 mg L^−1^ BA, significantly higher than all other treatments. TDZ, at all concentrations (0.1–2.0 mg L^−1^), failed to induce shoots (Figure 6). Leaf formation followed a similar trend. The greatest leaf number (2.5 leaves per protocorm) was also recorded with 1.0 mg L^−1^ BA, followed by 0.5 mg L^−1^ BA (1.5 leaves per protocorm), both significantly higher than the control. BA at 0.1 and 2.0 mg L^−1^ resulted in significantly fewer leaves per protocorm (0.8 and 0.7 leaves, respectively) compared to the control. In contrast, all kinetin treatments (0.1–2.0 mg L^−1^) produced 0.9–1.1 leaves per protocorm, with no significant difference from the control. TDZ did not induce leaf formation. Root induction showed a distinct response. The highest number of roots (4.2 leaves per protocorm) was observed with 1.0 mg L^−1^ Kinetin, which was significantly higher than the control (2.3 leaves). Rooting declined sharply at 2.0 mg L^−1^ BA (0.8 roots per protocorm). Rhizome formation was highest in the control treatment (1.4 rhizomes per protocorm). In contrast, 2.0 mg L^−1^ BA and kinetin significantly reduced rhizome formation, with the lowest number observed at 0.8 rhizomes per protocorm. Kinetin supported moderate rhizome formation, peaking at 0.1 mg L^−1^ (1.3 rhizomes per protocorm), but its effect declined at higher concentrations. However, TDZ did not induce rhizome formation at any tested concentration.

### 3.8. Effect of Auxins on Survival Rate, Callus Induction, Shoot and Root Formation, and Rhizome Development in Protocorms

**Figure 7:**
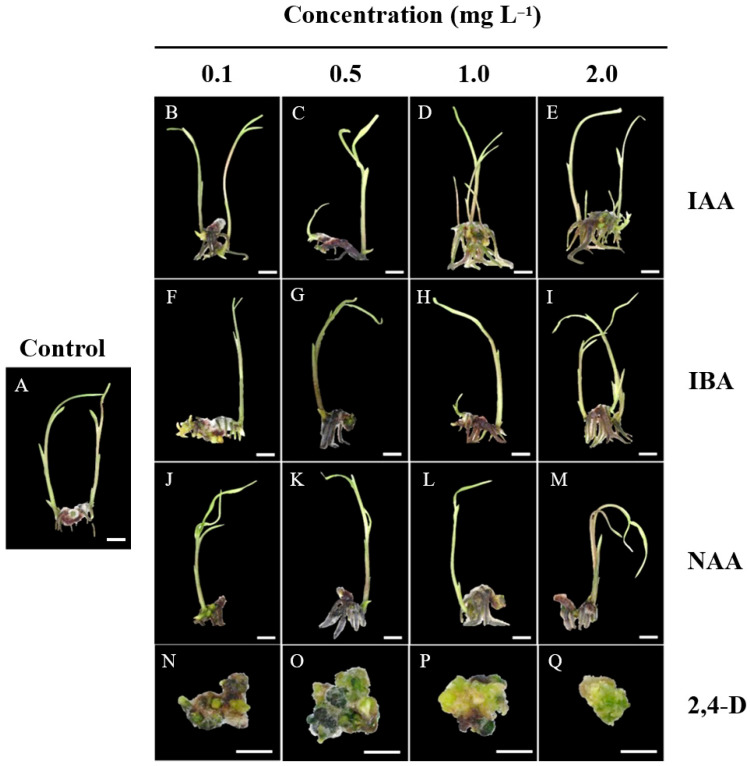
Growth responses of *E*. *bicallosa* protocorms on ½MS medium supplemented with auxins (IAA, IBA, NAA, and 2,4-D) at 0.1, 0.5, 1.0, and 2.0 mg L^−1^ after 12 weeks of culture. (**A**) Control (no auxin); (**B**–**E**) IAA enhanced shoot, root, and rhizome formation; (**F**–**I**) IBA promoted root and shoot development; (**J**–**M**) NAA resulted in moderate growth; (**N**–**Q**) 2,4-D inhibited shoot and root formation. Scale bar = 1 cm.

IAA treatments at 0.1 mg L^−1^ resulted in the highest survival rates across all concentrations, peaking at 96.0% with no significant between treatments, while increasing 2,4-D from (0.1–2.0 mg L^−1^) had the most detrimental impact, reducing survival from 60.0% (0.1 mg L^−1^) to 33.3% (2.0 mg L^−1^) (Table 8 and Figure 7).

In case of callus formation was absent in control, IAA, IBA, and NAA treatments but strongly induced by 2,4-D, with a peak (100.0%) at 0.1 mg L^−1^, decreasing to 73.3% at 2.0 mg L^−1^. Shoot formation was highest in IBA 0.5 mg L^−1^ (60.0%) but shoot formation declined 1.9 time as the concentrations IBA increased to 2.0 mg L^−1^ (30.8%). On the other hand, all 2,4-D concentration completely inhibited shoot formation (0.0%) (Table 8 and Figure 7). Concerning for root formation was low across treatments, with IBA (0.5–1.0 mg L^−1^) and NAA (0.5 mg L^−1^) achieving the highest rate (12.5%) while 2,4-D completely inhibited root development.

Rhizome formation occurred only in IAA, IBA, and NAA treatments, with IAA (1.0 mg L^−1^) exhibit the highest rhizome formation (41.7%), followed by IBA (35.4% at 1.0 mg L^−1^) with no significant between treatments. NAA had a weaker effect, with a maximum of 23.0% (0.5 mg L^−1^), declining at higher concentrations. 2,4-D did not promote rhizome formation.

### 3.9. Effect of Auxins on the Number of Shoots, Roots, and Rhizomes of Protocorms

The addition of IAA at 0.1 mg L^−1^ promoted the highest shoot number (1.1 shoots per explant) with non-significant difference among treatments, while higher concentrations (0.5–2.0 mg L^−1^) led to a decline in number of shoots (0.5–0.6 shoots per explant). In contrast, 2,4-D significantly inhibited shoot formation at concentration of 0.5–2.0 mg L^−1^ (Table 9).

For root formation, 1.0 mg L^−1^ IAA was the most effective (4.2 roots per explant). Higher concentrations of NAA reduced root formation, with a significant decline at 2.0 mg L^−1^ (0.6 roots per explant). Moreover, 2,4-D at 0.5–2.0 mg L^−1^ completely inhibited root induction (Table 9). Rhizome formation was highest at 0.1–0.5 mg L^−1^ IAA (1.5 rhizomes per explant). However, as the IAA increase from 1.0 to 2.0 mg L^−1^, rhizome formation declined significantly. IBA and NAA had minimal effects on rhizome induction, with results similar to the control. While 2,4-D showed a moderate increase at 0.5 mg L^−1^ (0.7 rhizomes per explant), higher concentrations (2.0 mg L^−1^) completely suppressed rhizome formation (Table 9).

### 3.10. Greenhouse Acclimatization of Plantlets

**Figure 8:**
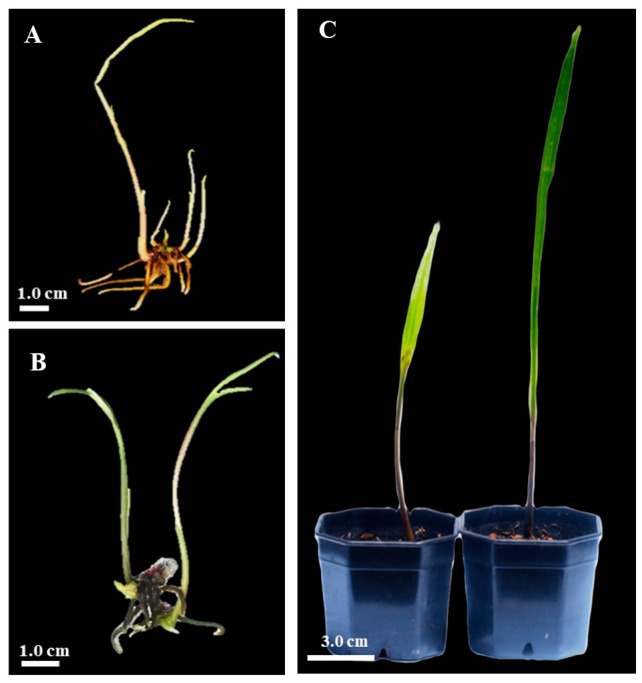
*E*. *bicallosa* plantlets derived from cytokinin ((**A**) ½MS + 1.0 mg L^−1^ BA) and auxin ((**B**) ½MS + 0.1 mg L^−1^ IAA) treatments, showing well-developed leaves, shoots, and roots, were selected for acclimatization in a greenhouse; (**C**) regenerated plantlets after 8 weeks of growth in plastic pots containing a 1:1:1 (*v*/*v*) mixture of pumice, sand, and loamy soil.

The in vitro-derived plantlets, displaying well-developed roots (Figure 8A,B), were successfully transferred to potting media consisting of pumice, sand, and soil in a 1:1:1 (*v/v*) ratio (Figure 8C). After 8 weeks in the greenhouse, the regenerated plantlets, with fully developed shoots and roots, achieved a 100% survival rate (Figure 8C).

## 4. Discussion

### 4.1. Impact of Light Quality on Seed Germination and Development

Light quality is a critical determinant in the germination and early development of orchid seeds. Due to their minute, non-endospermic nature, orchid seeds rely heavily on external environmental cues for successful germination [51,52]. Among these, the spectral quality of light significantly affects the transition from seed to protocorm a vital intermediate stage preceding seedling formation. Specific wavelengths can stimulate or suppress physiological and morphological changes necessary for protocorm development, making light quality a pivotal factor in optimizing in vitro propagation protocols for orchids. In the present study, all light treatments facilitated seed germination; however, blue and red light significantly accelerated the developmental progression of *E*. *bicallosa* seeds, enabling them to reach stage 5 within 24 weeks. This enhancement is attributed to light’s regulatory role in plant morphogenesis and photosynthetic activity, particularly under in vitro conditions [53,54].

Plants detect and respond to light through specific photoreceptors: cryptochromes (CRYs) perceive blue light, whereas phytochromes (PHYs) sense red (R) and far-red (FR) light. These photoreceptors orchestrate a broad range of developmental processes, including seed germination, seedling growth, and root initiation [55,56]. Consistent with our findings, Biswal et al. [57] demonstrated that blue light enhances seed germination and increases photosynthetic pigment accumulation, while red light promotes shoot and root elongation in *Dendrobium* ‘Sonia’. Similarly, Favetta et al. [58] reported that blue (460 nm) and red (640 nm) LED light significantly improved shoot proliferation and survival rates in *Microlaelia lundii*. Moreover, blue light has been shown to promote embryo enlargement and advance protocorm development in *Epipactis veratifolia* and *Dactylorhiza umberosa* [23]. In contrast, the lowest percentages of early-stage development in *E*. *bicallosa* were observed under dark conditions. This observation aligns with Lin et al. [59], who reported that darkness suppressed growth, stem formation, and chlorophyll content in protocorm-like bodies (PLBs) of *Dendrobium officinale*. Mahdavi et al. [60] also found that seeds of *Phalaenopsis amabilis* ‘Anthura Beijing’ stored in darkness had the lowest total germination rate (48.33%). Conversely, Hussien et al. [61] reported that dark conditions promoted protocorm development and seedling formation in *Dactylorhiza fuchsii*. These contrasting findings underscore that light responses in orchid seed germination are species-specific. Different wavelengths of light elicit distinct developmental outcomes, reinforcing the importance of tailoring light conditions to the physiological requirements of each species.

### 4.2. Effect of Culture Media on Survival and Morphological Responses of Protocorms

The composition, type, concentration, and ratio of macronutrients and micronutrients in the culture medium are critical factors that influence various stages of orchid protocorm development [19]. For *E*. *bicallosa* protocorms, ½MS medium was found to be the most effective in enhancing survival, growth, and morphological development compared to other tested media. This superior performance is likely attributed to its balanced supply of essential macro- (particularly nitrogen) and micronutrients, which are fundamental for key physiological processes, such as chlorophyll synthesis, protein production, and biomass accumulation [62,63]. Additionally, the reduced ionic strength of ½MS medium may help to mitigate osmotic stress while still providing sufficient nutrients, a condition particularly advantageous for orchids, which are naturally adapted to nutrient-deficient environments [64]. These findings are consistent with the results of Decruse et al. [65], who reported that ½MS medium significantly promoted rhizome formation and shoot development in *E*. *cullenii*. Similarly, Romeida et al. [66] observed that ½MS medium facilitated the highest proliferation of protocorm-like bodies (PLBs) in *Dendrobium* ‘Gatton Sunray’. In contrast, other media such as BM-1, VW, KC, and MM appeared less effective, possibly due to suboptimal nutrient concentrations or imbalanced nutrient ratios. The nutrient demands of orchids are species-specific, and optimal growth depends on both the concentration and chemical form of elements like nitrogen (N) and phosphorus (P) [67,68,69]. It is therefore essential to recognize that each orchid species has unique nutritional requirements, and its developmental response is closely tied to the specific composition of the culture medium [70,71]. For example, full-strength MS medium was reported as the most effective for promoting PLBs formation in *E*. *graminea* [72], while KC medium was identified as optimal for both seed germination and early development in *Cattleya nobilior* [73] as well as for seedling multiplication in *Oeceoclades maculata* [74]. These findings underscore the importance of selecting culture media that are tailored to the species-specific and developmental requirements in order to achieve successful in vitro propagation and conservation of orchids.

### 4.3. Influence of Coconut Water and Potato Extract on Protocorm Growth

The effects of organic additives, including coconut water (CW) and potato extract (PE), on the growth and development of *E*. *bicallosa* protocorms were investigated. The results demonstrated that the combination of CW and PE, particularly at concentrations of 150–200 mL L^−1^ CW and 25–50 g L^−1^ PE, significantly enhanced protocorm survival, morphogenesis, and the induction of shoots, leaves, roots, and rhizomes. Additionally, the application of PE alone at 25–50 g L^−1^ was found to promote shoot and root formation. However, the synergistic effect of CW and PE yielded superior outcomes, as media supplemented with 150–200 mL L^−1^ CW and 25–50 g L^−1^ PE led to a marked increase in the number of shoots, leaves, roots, and rhizomes per protocorm. This response may be attributed to the diverse range of minerals, sugars, and bioactive compounds present in coconut water, including plant growth regulators such as auxins and cytokinins, as well as vitamins (e.g., myo-inositol) and certain enzymes [75,76]. These components play essential roles in plant growth by stimulating embryo development and promoting cell division and organ formation [77,78]. Similarly, potato extract contributes significantly to protocorm growth, as it is rich in carbohydrates, amino acids, essential vitamins (C, B_1_, B_6_), and minerals such as potassium, iron, and magnesium [79,80]. Moreover, PE contains biosynthetic enzymes and polyamines that promote nucleic acid synthesis and enhance mitotic activity in plant tissues [81,82].

The beneficial effects of CW and PE have also been demonstrated in other orchid species. For instance, Barua et al. [83] reported that PE enhanced both shoot and root development in *Dendrobium aduncum*. In *D*. *cruentum*, Samala and Thipwong [84] observed that media supplemented with CW and PE significantly promoted protocorm formation and subsequent plantlet development compared to control media. Similarly, Laily et al. [85] found that the addition of 150 mL L^−1^ CW to the germination medium enhanced protocorm development and was effective for PLBs induction in *Vanda celebica*.

### 4.4. Effect of Cytokinins on Survival Rate, Callus Induction, Shoot and Root Formation, and Rhizome Development

Cytokinins play a crucial role in regulating plant developmental processes by modulating cell division, differentiation, and overall growth [86]. These plant growth regulators are particularly important in orchid protocorm development, where they influence shoot initiation, callus induction, leaf expansion, root formation, and rhizome development [87]. In the present study, BA demonstrated significant effects at varying concentrations. Low concentrations of BA (0.1 mg L^−1^) enhanced survival rates and rhizome formation, whereas 1.0 mg L^−1^ was most effective for promoting shoot formation and increasing the number of shoots and leaves. These effects are likely due to BA’s ability to stimulate cell division, elongation, and differentiation, particularly within the apical and axillary meristems, contributing to overall plant growth and development [88,89]. Moreover, BA regulates the expression of genes critical for shoot apical meristem activity, resulting in new shoot development and enhanced cell proliferation and expansion necessary for leaf formation [90,91]. In various orchid species, including *Eulophia*, BA has been shown to effectively promote shoot and leaf development. For example, Panwar et al. [48] reported that MS medium supplemented with 1.0 mg L^−1^ BA was optimal for shoot induction in *E*. *nuda*. Similarly, Dawande and Gurav [92] demonstrated that BA resulted in the highest percentage of shoot emergence from rhizomes in *E*. *nuda*, outperforming other cytokinins. In *Phalaenopsis* ‘P21-L-34-1033’, BA was also effective in stimulating both shoot and leaf formation [93].

Root formation was most enhanced at lower concentrations of kinetin (0.1 mg L^−1^), with the highest number of roots (4.2 per protocorm) recorded at 1.0 mg L^−1^. While cytokinins are often considered negative regulators of root development, low concentrations can promote adventitious root (AR) formation by encouraging cell division and differentiation [41,94]. These findings are consistent with those of Nguyen et al. [95], who demonstrated that kinetin supplementation promoted root formation from protocorms of *Dendrobium anosmum*. Similarly, in *D*. *stratiotes* plantlets, kinetin stimulated the highest number of roots [96]. In contrast, TDZ primarily induced callus formation, while inhibiting shoot, root, and rhizome development. TDZ is known for its strong influence on cell division, enlargement, and morphogenesis in vitro, which often leads to callus formation rather than organogenesis. For example, TDZ in combination with BA and NAA efficiently induced callus from internode, node, and leaf explants of *Anoectochilus elatus* [97]. Similarly, Handini et al. [98] reported that TDZ-supplemented medium was optimal for callus induction in *Paphioedilum glaucophyllum.*

### 4.5. Effect of Auxins on Survival Rate, Callus Induction, Shoot and Root Formation, and Rhizome Development

In this study, IAA, IBA, and NAA exhibited the most favorable effects on shoot, root and rhizome formation, whereas 2,4-D consistently inhibited development at across all tested concentrations. Specifically, 0.1 mg L^−1^ IAA yielded the highest average number of shoots, while 1.0 mg L^−1^ IAA promoted the greatest root development. A moderate concentration of 0.5 mg L^−1^ IBA was particularly effective in maintaining high survival rates while supporting overall plantlet growth. This concentration promoted shoot emergence (60.0%) and was also highly effective in root formation (12.5%). These results are consistent with previous studies, such as those by Panwar et al. [48] and Khumalo et al. [49], which demonstrated the effectiveness of IBA in inducing root formation in species of *Eulophia*, including *E*. *nuda* and *E*. *streptopetala*.

Root induction and development were notably influenced by the presence of IAA, IBA, and NAA in the MS medium. The application of auxins was particularly effective in promoting immediate root induction and subsequent root development of in the protocorms. Similarly, Guo et al. [99] reported that treatment with 0.5 mg L^−1^ NAA resulted in the longest average root length (2.06 cm) and the highest average number of roots (3.22 roots) of *Paphiopedilum* SCBG Huihuang90. However, higher NAA concentrations (1.0–2.0 mg L^−1^) did not enhance rooting rates or root numbers; instead, they inhibited root development. This inhibition may be due to excessive auxin accumulation, which disrupts hormonal balance within the explants. High auxin levels can induce stress, impairing essential cellular processes such as cell division and elongation, ultimately leading to reduced root growth [100].

Additionally, all concentrations of 2,4-D tested in this study effectively induced callus formation in *E*. *bicallosa* protocorms. This is consistent with the findings of Wang and Tian [101], who reported that 2,4-D resulted in the highest frequency of callus formation (48.0%) in *Bletilla striata*. Conversely, Warner et al. [102] found 2,4-D to be ineffective in inducing callus formation in *Vanilla odorata* and *V*. *pompona*.

The callus formation observed in *E*. *bicallosa* protocorms can be attributed to the well-known properties of 2,4-D as a potent auxin. 2,4-D is recognized for its ability to induce dedifferentiation, cell division, and expansion by regulating protein synthesis, which in turn affects enzyme activity, respiration, and overall cell division [103,104]. Exogenous auxins like 2,4-D are crucial in callus formation across many orchid species, including *Papilionanthe* ‘Miss Joaquim’ [105], *Vanilla planifolia* [106], *Dendrobium discolor* [107], and *Phalaenopsis circus* [108].

However, it was noted that increasing the concentration of 2,4-D in the culture medium led to higher protocorm mortality, with minimal or no development of shoots, roots, or other morphological features. This adverse effect may be linked to the herbicidal properties of 2,4-D, as higher concentrations have been shown to induce tissue necrosis and damage in plants [109].

### 4.6. Transplantation and Survival of Plantlets

The 100% survival rate of in vitro-derived *E*. *bicallosa* plantlets after 8 weeks of greenhouse acclimatization highlights the effectiveness of the developed protocol. The use of a 1:1:1 (*v*/*v*) mixture of pumice, sand, and loamy soil provided favorable aeration and drainage, supporting root development and minimizing transplant shock. Similar substrates have been reported to enhance survival in other orchid species by facilitating water retention and gas exchange [110,111]. The presence of well-developed roots and shoots at the time of transfer likely played a key role in this success, as strong root systems improve water uptake and anchorage under ex vitro conditions [112]. These findings confirm the suitability of this acclimatization approach for large-scale propagation and conservation of *E*. *bicallosa*.

## 5. Conclusions

This study presents an efficient in vitro propagation protocol for *E*. *bicallosa*, a rare Thai orchid species, by integrating asymbiotic seed germination with protocorm development. Light quality significantly influenced seedling development, with red and blue light promoting progression to more advanced stages, whereas darkness and white light were less effective. Among the tested media, half-strength MS medium was the most suitable, supporting superior protocorm growth and survival. The addition of organic supplements such as coconut water and potato extract, further enhanced shoot, leaf, and root development. Applying specific cytokinins and auxins individually, rather than in combination, allowed targeted promotion of either shoot or root formation, depending on the type and concentration of the plant growth regulator. Acclimatization using a soil-based substrate under greenhouse conditions resulted in high survival.

In conclusion, this protocol provides a reliable and scalable strategy for rapid clonal propagation and ex situ conservation of *E*. *bicallosa*, with strong potential to support future restoration and reintroduction efforts for this and other threatened orchid species.

## Figures and Tables

**Table 1:** Seed germination and seedling developmental stages of *E*. *bicallosa*.

Developmental Stage	Description
0	Hyaline embryo with intact testa
1	Swollen embryo, ruptured testa, and emergence of rhizoids (germination)
2	Appearance of protomeristem
3	Formation of a scale leaf and a rhizome-like structure at the base of the promeristem in the protocorm
4	Continued enlargement of the rhizome-like structure and/or emergence of the first leaf from rhizome-like structure
5	Fully developed leaf from shoots-derived protocorms (seedling)

**Table 2:** Effect of different culture media on the percentage of survival, shoot, root, rhizome, and callus formation of *E*. *bicallosa* protocorms after 12 weeks of culture.

Culture Media	Percentage (%)				
	Survival	Shoot Formation	Rooting	Rhizome	Callus
½MS	100.0 ± 0.0 ns	93.3 ± 3.3 a	96.7 ± 3.3 a	100.0 ± 0.0 a	0.0 ± 0.0 b
MS	90.0 ± 0.0	36.7 ± 8.8 b	43.3 ± 8.8 bc	50.0 ± 5.8 c	26.7 ± 3.3 a
VW	86.7 ± 6.7	30.0 ± 0.0 b	40.0 ± 5.8 c	80.0 ± 0.0 ab	0.0 ± 0.0 b
BM-1	96.7 ± 3.3	0.0 ± 0.0 c	43.3 ± 3.3 bc	3.3 ± 3.3 d	0.0 ± 0.0 b
KC	96.7 ± 3.3	30.0 ± 5.8 b	63.3 ± 3.3 b	90.0 ± 5.8 ab	0.0 ± 0.0 b
MM	86.7 ± 6.7	0.0 ± 0.0 c	63.3 ± 8.8 b	66.7 ± 6.7 bc	0.0 ± 0.0 b

Results are mean of 3 replicates (30 protocorms per replicate). Error bars represent the standard error (SE), and different letters on each bar indicate significant differences at *p* ≤ 0.05 according to DMRT, ns, no significance.

**Table 3:** Effect of different culture media on the number of shoots, leaves, roots, and rhizomes in *E*. *bicallosa* protocorms after 12 weeks of culture.

Culture Media	Number per Protocorm			
	Shoots	Leaves	Roots	Rhizome
½MS	1.0 ± 0.0 a	1.1 ± 0.1 a	4.2 ± 0.1 a	2.1 ± 0.4 ns
MS	0.5 ± 0.1 b	0.5 ± 0.1 b	1.9 ± 0.5 b	1.6 ± 0.4
VW	0.3 ± 0.0 b	0.3 ± 0.0 b	1.2 ± 0.1 bc	2.2 ± 0.4
BM-1	0.0 ± 0.0 c	0.0 ± 0.0 c	0.6 ± 0.1 c	2.3 ± 0.3
KC	0.5 ± 0.2 b	0.5 ± 0.1 b	1.7 ± 0.2 b	1.8 ± 0.2
MM	0.0 ± 0.0 c	0.0 ± 0.0 c	1.3 ± 0.1 bc	1.4 ± 0.0

Results are mean of 3 replicates (30 protocorms per replicate). Error bars represent the standard error (SE), and different letters on each bar indicate significant differences at *p* ≤ 0.05 according to DMRT, ns, no significance.

**Table 4:** Effect of coconut water and potato extract on survival, shoot formation, rooting, and rhizome formation of *E*. *bicallosa* protocorms after 12 weeks of culture.

CW ^1^ (mL L^−1^)	PE ^2^ (g L^−1^)	Survival Rate (%)	Shoot Formation (%)	Rooting (%)	Rhizome Formation (%)
0	0	86.7 ± 3.3 b	46.7 ± 6.7 c–g	53.3 ± 2.3 b–d	80.0 ± 5.8 cd
0	25	96.7 ± 3.3 ab	80.0 ± 0.0 a	90.0 ± 0.0 a	96.7 ± 3.3 ab
0	50	100.0 ± 0.0 a	76.7 ± 8.8 ab	90.0 ± 5.8 a	100.0 ± 0.0 a
0	100	100.0 ± 0.0 a	43.3 ± 3.3 d–g	53.3 ± 6.7 b–d	96.7 ± 3.3 ab
50	0	96.7 ± 3.3 ab	63.3 ± 8.8 a–f	63.3 ± 6.7 a–d	90.0 ± 0.0 a–d
100	0	86.7 ± 8.8 b	40.0 ± 7.3 e–g	36.7 ± 4.5 d	83.3 ± 8.8 b–d
150	0	93.3 ± 3.3 ab	40.0 ± 5.3 e–g	46.7 ± 3.3 cd	90.0 ± 5.8 a–d
200	0	96.7 ± 3.3 ab	30.0 ± 2.3 g	36.7 ± 2.3 d	86.7 ± 8.8 a–d
50	25	100.0 ± 0.0 a	66.7 ± 3.3 a–e	83.3 ± 2.0 ab	96.7 ± 3.3 ab
100	25	96.7 ± 3.3 ab	73.3 ± 8.8 a–c	83.3 ± 6.7 ab	96.7 ± 3.3 ab
150	25	100.0 ± 0.0 a	70.0 ± 5.8 a–d	83.3 ± 3.3 ab	100.0 ± 0.0 a
200	25	96.7 ± 3.3 ab	63.3 ± 6.7 a–f	66.7 ± 8.8 a–d	93.3 ± 3.3 a–c
50	50	96.7 ± 3.3 ab	73.3 ± 6.7 a–c	80.0 ± 5.8 ab	96.7 ± 3.3 ab
100	50	96.7 ± 3.3 ab	53.3 ± 6.7 a–g	60.0 ± 5.8 a–d	90.0 ± 5.8 a–d
150	50	100.0 ± 0.0 a	76.7 ± 6.7 ab	83.3 ± 8.8 ab	96.7 ± 3.3 ab
200	50	93.3 ± 3.3 ab	70.0 ± 5.8 a–d	70.0 ± 5.8 a–c	93.3 ± 3.3 a–c
50	100	100.0 ± 0.0 a	50.0 ± 0.0 b–g	60.0 ± 0.0 a–d	100.0 ± 0.0 a
100	100	93.3 ± 3.3 ab	40.0 ± 5.8 e–g	56.7 ± 3.3 b–d	86.7 ± 3.3 a–d
150	100	100.0 ± 0.0 a	36.7 ± 6.7 fg	40.0 ± 5.8 cd	96.7 ± 3.3 ab
200	100	86.7 ± 3.3 b	43.3 ± 2.0 d–g	46.7 ± 3.6 cd	76.7 ± 6.7 d

Results are mean of 3 replicates (30 protocorms per replicate). Error bars represent the standard error (SE), and different letters on each bar indicate significant differences at *p* ≤ 0.05 according to DMRT, ns, no significance. ^1^ CW, coconut water. ^2^ PE, potato extract.

**Table 5:** Effect of coconut water and potato extract on shoots, leaves, roots, and rhizomes induction number in *E*. *bicallosa* protocorms after culture for 12 weeks.

CW ^1^ (mL L^−1^)	PE ^2^ (g L^−1^)	Number per Protocorm			
		Shoots	Leaves	Roots	Rhizomes
0	0	0.9 ± 0.2 b–e	1.0 ± 0.2 b–e	3.3 ± 0.8 def	1.2 ± 0.4 e
0	25	1.3 ± 0.1 a–e	1.1 ± 0.1 b–e	5.8 ± 0.2 a–e	1.7 ± 0.3 b–e
0	50	1.0 ± 0.2 b–e	1.0 ± 0.2 b–e	5.9 ± 0.8 a–d	1.4 ± 0.1 de
0	100	0.6 ± 0.1 e	0.6 ± 0.1 e	2.3 ± 0.4 f	1.6 ± 0.2 cde
50	0	1.3 ± 0.2 a–e	1.0 ± 0.3 b–e	3.8 ± 0.3 c–f	2.3 ± 0.2 abc
100	0	0.9 ± 0.4 cde	0.8 ± 0.3 de	2.5 ± 1.1 f	2.5 ± 0.4 abc
150	0	0.9 ± 0.3 cde	0.8 ± 0.2 cde	2.7 ± 0.9 ef	2.8 ± 0.5 a
200	0	0.7 ± 0.4 de	0.8 ± 0.5 de	2.6 ± 1.6 f	2.5 ± 0.1 ab
50	25	1.2 ± 0.1 a–e	1.4 ± 0.2 a–e	7.0 ± 1.3 ab	2.1 ± 0.1 a–d
100	25	1.5 ± 0.2 abc	1.6 ± 0.2 a–d	7.2 ± 1.1 ab	2.3 ± 0.2 abc
150	25	1.4 ± 0.2 a–d	1.7 ± 0.2 ab	6.7 ± 0.6 abc	2.7 ± 0.6 a
200	25	1.5 ± 0.2 abc	1.8 ± 0.4 ab	6.6 ± 1.7 abc	2.6 ± 0.2 ab
50	50	1.3 ± 0.1 a–e	1.6 ± 0.3 abc	5.9 ± 0.5 a–d	2.7 ± 0.1 a
100	50	0.9 ± 0.2 cde	1.0 ± 0.3 b–f	4.7 ± 1.0 b–f	2.2 ± 0.1 a–d
150	50	1.6 ± 0.1 ab	1.9 ± 0.1 a	8.8 ± 1.0 a	2.6 ± 0.2 ab
200	50	1.7 ± 0.1 a	1.7 ± 0.1 ab	7.3 ± 0.7 ab	2.7 ± 0.1 a
50	100	0.8 ± 0.1 de	0.9 ± 0.1 cde	4.4 ± 0.2 b–f	2.2 ± 0.1 a–d
100	100	0.8 ± 0.1 de	0.8 ± 0.1 cde	3.7 ± 0.4 c–f	2.4 ± 0.2 abc
150	100	0.7 ± 0.1 de	0.7 ± 0.1 e	2.5 ± 0.4 f	2.4 ± 0.3 abc
200	100	0.9 ± 0.4 cde	0.8 ± 0.4 cde	4.1 ± 1.7 b–f	2.5 ± 0.3 abc

Results are mean of 3 replicates (30 protocorms per replicate). Error bars represent the standard error (SE), and different letters on each bar indicate significant differences at *p* ≤ 0.05 according to DMRT. ^1^ CW, coconut water. ^2^ PE, potato extract.

**Table 6:** Effects of cytokinins on survival, callus induction, and the formation of shoots, roots, and rhizomes in *E*. *bicallosa* protocorms after 12 weeks of culture.

Cytokinin	Concentration (mg L^−1^)	Survival Rate (%)	Callus Induction (%)	Shoot Formation (%)	Rooting (%)	Rhizome Formation (%)
Control	0.0	73.0 ± 4.0 d	0.0 ± 0.0 c	79.0 ± 2.0 b	60.3 ± 7.5 ab	77.0 ± 4.0 ab
BA	0.1	96.0 ± 2.0 a	0.0 ± 0.0 c	87.7 ± 9.5 ab	45.7 ± 10.3 bc	81.3 ± 7.2 a
	0.5	85.3 ± 4.3 a–d	0.0 ± 0.0 c	92.0 ± 2.0 a	56.3 ± 3.8 ab	73.0 ± 5.3 ab
	1.0	92.0 ± 2.0 ab	0.0 ± 0.0 c	93.0 ± 3.3 a	62.7 ± 3.8 a	69.0 ± 0.0 abc
	2.0	77.3 ± 5.6 cd	0.0 ± 0.0 c	77.3 ± 5.6 b	35.3 ± 7.5 c	45.7 ± 7.5 d
Kinetin	0.1	89.7 ± 4.3 abc	0.0 ± 0.0 c	87.7 ± 3.8 ab	67.0 ± 4.0 a	73.0 ± 4.0 ab
	0.5	88.0 ± 0.0 abc	0.0 ± 0.0 c	88.0 ± 0.0 ab	58.7 ± 7.5 ab	73.0 ± 5.3 ab
	1.0	85.7 ± 2.3 abc	0.0 ± 0.0 c	83.3 ± 2.3 ab	57.3 ± 1.3 ab	54.3 ± 11.8 cd
	2.0	83.3 ± 2.3 bcd	0.0 ± 0.0 c	85.7 ± 2.3 ab	54.0 ± 2.0 ab	60.0 ± 5.5 bcd
TDZ	0.1	85.3 ± 4.3 a–d	90.0 ± 0.0 b	0.0 ± 0.0 c	0.0 ± 0.0 d	0.0 ± 0.0 e
	0.5	81.3 ± 7.2 bcd	90.0 ± 0.0 b	0.0 ± 0.0 c	0.0 ± 0.0 d	0.0 ± 0.0 e
	1.0	83.3 ± 2.3 bcd	93.3 ± 3.3 a	0.0 ± 0.0 c	0.0 ± 0.0 d	0.0 ± 0.0 e
	2.0	79.0 ± 2.0 cd	90.0 ± 0.0 b	0.0 ± 0.0 c	0.0 ± 0.0 d	0.0 ± 0.0 e

Results are mean of 3 replicates (30 protocorms per replicate). Error bars represent the standard error (SE), and different letters on each bar indicate significant differences at *p* ≤ 0.05 according to DMRT.

**Table 7:** Effects of cytokinins on the number of shoots, leaves, roots, and rhizomes in *E*. *bicallosa* protocorms after 12 weeks of culture.

Cytokinin	Concentration (mg L^−1^)	Number per Protocorm			
		Shoots	Leaves	Roots	Rhizome
Control	0.0	0.9 ± 0.1 bc	1.0 ± 0.2 ab	2.3 ± 0.5 bcd	1.4 ± 0.3 a
BA	0.1	0.8 ± 0.2 bc	0.8 ± 0.1 c	1.4 ± 0.9 cde	0.9 ± 0.3 abc
	0.5	1.3 ± 0.2 b	1.5 ± 0.2 b	1.9 ± 0.2 cd	1.0 ± 0.1 abc
	1.0	2.3 ± 0.3 a	2.5 ± 0.2 a	2.8 ± 0.5 abc	1.2 ± 0.1 abc
	2.0	0.9 ± 0.0 bc	0.7 ± 0.3 c	0.8 ± 0.3 de	0.8 ± 0.1 c
Kinetin	0.1	1.0 ± 0.3 bc	1.1 ± 0.3 ab	4.0 ± 0.7 a	1.3 ± 0.3 ab
	0.5	0.9 ± 0.1 bc	1.0 ± 0.1 ab	4.0 ± 0.4 a	0.9 ± 0.0 bc
	1.0	0.8 ± 0.2 bc	0.9 ± 0.2 ab	4.2 ± 0.7 a	1.0 ± 0.0 abc
	2.0	0.7 ± 0.2 bc	0.9 ± 0.2 ab	3.6 ±0.6 ab	0.8 ± 0.1 c
TDZ	0.1	0.0 ± 0.0 d	0.0 ± 0.0 d	0.0 ± 0.0 e	0.0 ± 0.0 d
	0.5	0.0 ± 0.0 d	0.0 ± 0.0 d	0.0 ± 0.0 e	0.0 ± 0.0 d
	1.0	0.0 ± 0.0 d	0.0 ± 0.0 d	0.0 ± 0.0 e	0.0 ± 0.0 d
	2.0	0.0 ± 0.0 d	0.0 ± 0.0 d	0.0 ± 0.0 e	0.0 ± 0.0 d

Results are mean of 3 replicates (30 protocorms per replicate). Error bars represent the standard error (SE), and different letters on each bar indicate significant differences at *p* ≤ 0.05 according to DMRT.

**Table 8:** Effects of auxins on survival, callus induction, and shoot, root, and rhizome formation in *E*. *bicallosa* protocorms after 12 weeks of culture.

Auxin	Concentration (mg L^−1^)	Survival Rate (%)	Callus Induction (%)	Shoot Formation (%)	Rooting (%)	Rhizome Formation (%)
Control	0.0	79.0 ± 2.0 cd	0.0 ± 0.0 c	55.0 ± 3.8 ab	6.3 ± 0.3 ab	0.0 ± 0.0 e
IAA	0.1	96.0 ± 2.0 a	0.0 ± 0.0 c	47.5 ± 6.5 abc	0.0 ± 0.0 b	0.0 ± 0.0 e
	0.5	85.3 ± 4.3 a–d	0.0 ± 0.0 c	51.7 ± 2.1 abc	8.4 ± 2.1 ab	16.7 ± 2.1 cde
	1.0	92.0 ± 2.0 ab	0.0 ± 0.0 c	55.8 ± 5.0 ab	6.3 ± 0.6 ab	41.7 ± 1.0 a
	2.0	77.3 ± 5.6 cd	0.0 ± 0.0 c	41.3 ± 3.6 abc	6.3 ± 0.7 ab	31.3 ± 6.2 abc
IBA	0.1	89.7 ± 4.3 a–c	0.0 ± 0.0 c	39.2 ± 2.1 abc	0.0 ± 0.0 b	0.0 ± 0.0 e
	0.5	88.0 ± 0.0 a–c	0.0 ± 0.0 c	60.0 ± 4.2 a	12.5 ± 2.2 a	22.9 ± 3.1 bcd
	1.0	85.7 ± 2.3 a–c	0.0 ± 0.0 c	41.3 ± 3.5 abc	12.5 ± 3.6 a	35.4 ± 2.7 ab
	2.0	83.3 ± 2.3 b–d	0.0 ± 0.0 c	30.8 ± 2.1 bcd	2.1 ± 2.1 ab	22.9 ± 2.1 bcd
NAA	0.1	73.0 ± 4.0 d	0.0 ± 0.0 c	43.3 ± 2.1 abc	2.1 ± 0.5 ab	0.0± 0.0 e
	0.5	81.3 ± 7.2 b–d	0.0 ± 0.0 c	43.3 ± 5.3 abc	12.5 ± 2.3 a	23.0 ± 4.2 bcd
	1.0	85.3 ± 4.3 a–d	0.0 ± 0.0 c	45.4 ± 4.1 abc	8.3 ± 1.2 ab	8.4 ± 2.1 de
	2.0	83.3 ± 2.3 b–d	0.0 ± 0.0 c	28.8 ± 7.2 cd	2.1 ± 2.1 ab	4.2 ± 4.2 e
2,4–D	0.1	60.0 ± 3.6 a–c	100.0 ± 0.0 a	0.0 ± 0.0 d	0.0 ± 0.0 b	0.0 ± 0.0 e
	0.5	78.8 ± 10.8 a–c	83.3 ± 1.5 ab	0.0 ± 0.0 d	0.0 ± 0.0 b	0.0 ± 0.0 e
	1.0	49.6 ± 16.3 cd	76.7 ± 0.6 b	0.0 ± 0.0 d	0.0 ± 0.0 b	0.0 ± 0.0 e
	2.0	33.3 ± 1.6 d	73.3 ± 0.6 b	0.0 ± 0.0 d	0.0 ± 0.0 b	0.0 ± 0.0 e

Results are mean of 3 replicates (30 protocorms per replicate). Error bars represent the standard error (SE), and different letters on each bar indicate significant differences at *p* ≤ 0.05 according to DMRT.

**Table 9:** Effect of auxins on the number of shoots, roots, and rhizomes in *E*. *bicallosa* protocorms after 12 weeks of culture.

Auxin	Concentration (mg L^−1^)	Number per Protocorm		
		Shoots	Roots	Rhizomes
Control	0.0	0.7 ± 0.1 bc	3.2 ± 0.7 a	0.8 ± 0.1 b
IAA	0.1	1.1 ± 0.1 a	3.0 ± 1.2 ab	1.5 ± 0.4 a
	0.5	0.5 ± 0.1 bcd	2.1 ± 0.4 abc	1.5 ± 0.4 a
	1.0	0.6 ± 0.2 bc	4.2 ± 1.6 a	0.6 ± 0.2 bc
	2.0	0.6 ± 0.1 bcd	2.6 ± 0.3 abc	0.5 ± 0.2 bc
IBA	0.1	0.6 ± 0.1 bc	2.4 ± 0.3 abc	0.2 ± 0.2 bc
	0.5	0.9 ± 0.1 ab	4.1 ± 0.2 a	0.6 ± 0.2 bc
	1.0	0.7 ± 0.2 bc	2.8 ± 1.0 ab	0.3 ± 0.1 bc
	2.0	0.6 ± 0.1 bc	2.9 ± 0.7 ab	0.4 ± 0.2 bc
NAA	0.1	0.4 ± 0.0 cde	2.2 ± 0.6 abc	0.3 ± 0.1 bc
	0.5	0.5 ± 0.2 bcd	2.1 ± 0.4 abc	0.3 ± 0.1 bc
	1.0	0.5 ± 0.1 bcd	1.0 ± 0.4 bcd	0.4 ± 0.1 bc
	2.0	0.2 ± 0.2 def	0.6 ± 0.4 cd	0.2 ± 0.1 bc
2,4–D	0.1	0.1 ± 0.1 ef	0.1 ± 0.1 d	0.5 ± 0.3 bc
	0.5	0.0 ± 0.0 f	0.0 ± 0.0 d	0.7 ± 0.1 bc
	1.0	0.0 ± 0.0 f	0.0 ± 0.0 d	0.6 ± 0.4 bc
	2.0	0.0 ± 0.0 f	0.0 ± 0.0 d	0.0 ± 0.0 c

Results are mean of 3 replicates (30 protocorms per replicate). Error bars represent the standard error (SE), and different letters on each bar indicate significant differences at *p* ≤ 0.05 according to DMRT.

## Data Availability

The data related to the findings of this research are available upon request from the corresponding author.

## Abbreviations

The following abbreviations are used in this manuscript:

CW	Coconut water
PE	Potato extract
